# Effects of auricular acupressure on polysomnography-assessed sleep, blood glucose, and stress in elderly patients with type 2 diabetes and insomnia: a randomized controlled trial

**DOI:** 10.3389/fpsyt.2025.1677240

**Published:** 2025-11-10

**Authors:** Jihye Nam, Hyejin Lee, Hyojung Park

**Affiliations:** Department of Nursing, Ewha Womans University, Seoul, Republic of Korea

**Keywords:** aged, blood glucose, diabetes mellitus type 2, sleep disorder, stress

## Abstract

**Objective:**

Elderly patients with type 2 diabetes frequently experience stress and sleep disorders, leading to poor glycemic control. Auricular acupressure, a non-pharmacological intervention, shows potential for improving blood glucose and sleep quality. This study aimed to comprehensively evaluate the combined effects of auricular acupressure on blood glucose, sleep, and stress, using objective measures.

**Methods:**

A randomized, single-blind, sham-controlled trial included 44 elderly individuals (65–85 years old) with type 2 diabetes and insomnia (PSQI ≥ 5). Participants were randomly assigned to an intervention or sham control group. The 8-week intervention involved specific therapeutic ear points. Sleep quality was assessed subjectively (PSQI) and objectively (polysomnography); postprandial blood glucose, weekly; and stress levels, via questionnaires (PSS, DASS-21) and brainwave analysis (EEG). Data underwent statistical analysis.

**Results:**

The intervention group demonstrated significant improvements in objective sleep measures. Specifically, deep sleep duration (SWS) significantly increased by 12.64% (|t|=2.585,*p* = 0.001), and sleep efficiency (SE) showed significant differences (|t|=2.354,*p* = 0.019). Postprandial blood glucose levels significantly decreased in the intervention group (F = 4.73,*p* = 0.032), while increasing in the sham control group. Objective stress indices from EEG also improved, with physical stress decreasing (left brain |t|=4.608,*p* < 0.001; right brain |t|=5.539,*p* < 0.001) and stress resistance increasing (left brain |t|=3.696,*p* < 0.001; right brain |t| =3.771,*p* < 0.001). However, no significant differences were observed in subjective stress scores between groups. No adverse events were found in either group during the whole study.

**Conclusion:**

Auricular acupressure is an effective and safe non-pharmacological intervention for improving objective sleep quality, lowering postprandial blood glucose, and reducing physiological stress in elderly patients with type 2 diabetes and insomnia.

**Clinical Trial Registration:**

https://cris.nih.go.kr/cris/search/detailSearch.do?seq=27425&search_page=M&search_lang=&class_yn=, identifier KCT0009524.

## 1 Introduction

Diabetes mellitus (DM) is a rapidly escalating global health concern, with projections indicating 1.3 billion affected individuals worldwide by 2050 (1). In South Korea, the prevalence among adults aged 60 and over reached 56.6% in 2021 (2). Elderly patients with type 2 diabetes (T2D) frequently experience diminished quality of life through macrovascular and microvascular complications, alongside psychosocial burdens including stress and sleep disorders (3). Diabetes-related stress affects up to 40% of patients, activating the hypothalamic-pituitary-adrenal (HPA) axis and negatively impacting blood glucose control (4). Concurrently, insomnia affects approximately 39% of elderly T2D patients (5), creating a vicious cycle of poor glycemic control as sleep critically influences glucose regulation and insulin sensitivity (6).

Non-pharmacological interventions are particularly vital for older adults to minimize risks associated with polypharmacy (7). Among complementary and alternative therapies, auricular acupressure has demonstrated potential for improving fasting blood glucose in T2D patients and enhancing sleep quality in the elderly (8–10). As a simple, non-invasive intervention, auricular acupressure offers advantages of safety, effectiveness, and accessibility (11). Recent studies by Lee, Kim, and Park (2025) (12) and Kim and Park (2024) (13) reported improvements in glycemic markers, stress, and sleep measures. However, these studies utilized assessment tools such as heart rate variability, Fitbit trackers, and actigraphy, which, while effective for general monitoring, offer limited scope for comprehensively elucidating specific neurophysiological pathways. Heart rate variability does not directly capture distinct brainwave patterns reflecting neural activity related to stress or sleep regulation, while actigraphy lacks the precision of polysomnography (PSG) to capture intricate sleep architecture crucial for evaluating restorative sleep.

The proposed mechanism of auricular acupressure involves stimulating specific auricular points that transmit signals to the central nervous system, potentially through the vagus nerve, influencing the hypothalamus and brainstem vital for sleep-wake regulation (14, 15). However, prior research has not precisely captured which specific brainwave patterns are modulated to produce these effects, leaving a crucial gap in understanding the full physiological mechanism.

Therefore, considering the intricate interconnections among blood glucose, sleep, and stress in elderly patients with T2D and insomnia, this study aims to rigorously evaluate auricular acupressure’s combined effects on glycemic control (specifically postprandial blood glucose), detailed objective sleep parameters (via PSG), and physiological stress responses (via EEG) in older adults with T2D and insomnia. This comprehensive objective assessment, particularly through direct analysis of brainwave patterns and intricate sleep architecture, will provide deeper insights into specific physiological mechanisms and robust clinical applicability of auricular acupressure for managing these interrelated health issues.

## 2 Methods

### 2.1 Study design

A randomized, single-blind, sham-controlled clinical trial was conducted to investigate the effects of auricular acupressure on blood glucose, stress, and sleep in elderly patients with type 2 diabetes. The aim was to investigate the effects of auricular acupressure on postprandial blood glucose, stress, and sleep in elderly patients with type 2 diabetes and insomnia. This trial was registered with the Clinical Research Information Service (CRiS), Republic of Korea, under registration number KCT0009524 on June 13, 2024. Participants were single-blinded to their group assignment. The primary outcomes focused on objective sleep quality and glucose control, reflecting the study’s emphasis on elderly patients with both type 2 diabetes and insomnia. Secondary outcomes included subjective measures and stress-related indicators to comprehensively evaluate the intervention’s broader physiological and psychological effects.

### 2.2 Participants and settings

Among the elderly who attended one of the four welfare facilities for the aged located in Seoul, Incheon, and Changwon, those who were diagnosed with type 2 diabetes and had sleep disorders were recruited from April 1 to June 2024. The 8-week intervention was conducted from June 15 to August 10, 2024. The specific criteria for subject selection and exclusion were as follows.

The criteria for the subject selection were the following: 1) aged 65 years or older; 2) treated with oral hypoglycemic agent or insulin since the diagnosis of type 2 diabetes; 3) conscious enough to complete the consent form and questionnaires; and 4) have sleep quality (PSQI) score of more than 5 points.

The criteria for the subject exclusion were the following: 1) currently taking sleeping pills; 2) have a mental illness (depression, anxiety disorder); 3) have a lesion on the ears or tape allergies; and 4) currently receiving other complementary-alternative therapies.

### 2.3 Sample size and randomization

**Figure 1:**
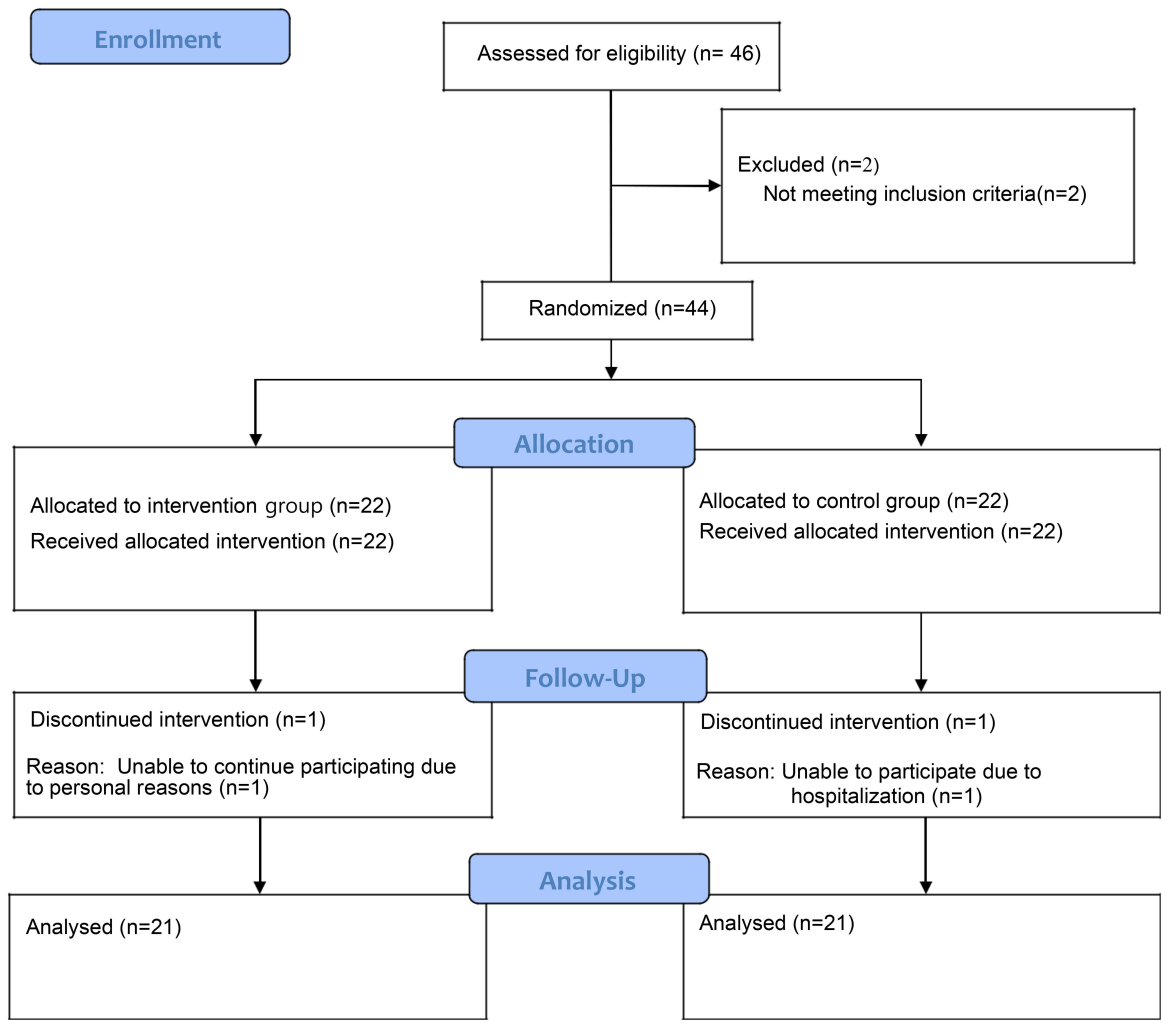
CONSORT flow diagram. This diagram illustrates the flow of participants through the randomized controlled trial, including recruitment, allocation, follow-up, and final analysis.

The sample size was calculated using G*Power 3.1 software. Based on previous studies, an independent sample t-test (two-tailed) indicated a minimum of 20 subjects per group, totaling 40 participants. The effect size, significance level, and power were set at 0.93, 0.05, and 0.80, respectively, considering a study on glycemic control in elderly patients with type 2 diabetes (effect size of 0.80, α = 0.05, 1-β = 0.80), a study on the effect of auricular acupressure on sleep among elderly postmenopausal women (effect size of 0.93, α = 0.05, 1-β = 0.80), and a meta-analysis on the effect of auricular acupressure on sleep in the elderly. Considering the eight-week study period and a 20% expected dropout rate, a total of 48 subjects (24 per group) were planned. In the recruitment process, 46 individuals voluntarily applied. Of these, 44 participants were finally enrolled after excluding 2 who met the exclusion criteria. For random assignment, participants were allocated to two groups using the RAND function of Excel, resulting in 22 patients assigned to the intervention group and 22 patients to the sham control group. To ensure allocation concealment, the random assignment sequence was generated in advance using the RAND function in Excel by an independent researcher who was not involved in participant recruitment or data collection. The assignment results were kept confidential and disclosed only after participants completed baseline assessments. While participants were single-blinded to their group assignment, the researchers applying the acupressure were not. However, to maintain the integrity of the statistical analysis, the data was provided to a separate statistical analyst who was completely blinded to the group assignments. Furthermore, the welfare facilities for the intervention and sham control groups were chosen to be geographically distant from each other (in Seoul and Changwon, respectively) to prevent any potential exposure or communication between the groups. During the study, one person from the intervention group and one person from the sham control group dropped out due to personal reasons. Ultimately, a total of 42 participants (21 in the intervention group and 21 in the sham control group) were included in the final analysis (**Figure 1**).

### 2.4 Intervention

**Figure 2:**
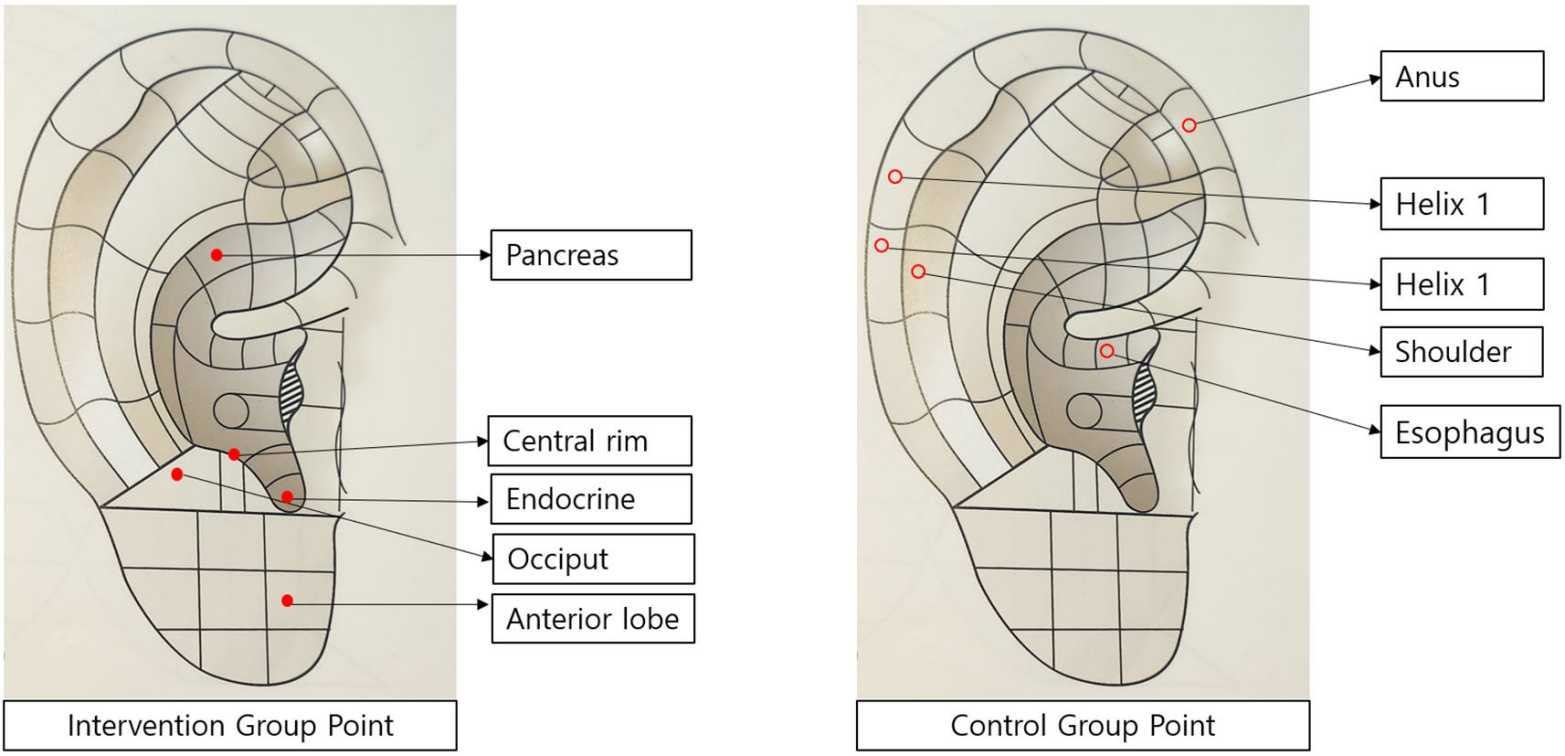
Auricular acupuncture points used in intervention and Sham Control Groups. Anatomical locations of the five therapeutic points used in the intervention group (marked as solid circles) (endocrine, central rim, occiput, anterior lobe, pancreas) and five non-therapeutic points used in the sham control group (marked as open circles) (shoulder, helix 1-2, esophagus, anus).

The reflex points for auricular acupressure were selected by analyzing a review of literature (8, 11, 13) and a previous study (9). The initial list was made focusing on the reflex points related to blood glucose, stress, and sleep improvement, and the final reflex points were determined after verification by one auricular acupressure expert. Through this, the validity of the reflex points to be applied to the intervention group and control group was secured. **Figure 2**. Auricular Acupuncture Points Used in Intervention and Sham Control Groups. Anatomical locations of the five therapeutic points used in the intervention group (marked as solid circles) (endocrine, central rim, occiput, anterior lobe, pancreas) and five non-therapeutic points used in the sham control group (marked as open circles) (shoulder, helix 1-2, esophagus, anus).Regarding the selected reflex points, the appropriateness and accuracy of positioning were verified by an auricular acupressure specialist. According to the analysis on previous studies, the duration of applying auricular acupressure and stimulation varied from 2~8 weeks, but it was found to be effective in most studies (8, 13, 16–18). In this study, it was decided to apply auricular acupressure for eight weeks to improve blood glucose and stress levels, based on the effective intervention periods consistently identified in meta-analyses of prior research.

Before the implementation of auricular acupressure, the foreign matters on the ears were removed by disinfecting with an alcohol pad and the general condition was assessed. Afterwards, a 1*1cm patch with a seed was applied to the reflex points of the ear. The subjects were instructed to press the patch-applied areas for 2~3 seconds at least three times a day. The patches were applied for five days a week, with alternating application to both ears every one week. Also, the subjects were instructed to remove the patches on the sixth day. In order to prevent the exposure of the study, the auricular acupressure was applied by meeting the subjects one-on-one or in the same group. Intervention fidelity was managed by sending text messages to participants one day prior to each weekly meeting, and by providing daily reminders for pressing the patches three times a day at consistent times, to ensure adherence to the intervention protocol. For the control group, all procedures except for the ear reflex points were performed in the same way as in the intervention group.

To enhance participant compliance with the intervention, several strategies were employed. First, on the initial day of the study, all participants received comprehensive education on how to perform self-acupressure. They were required to demonstrate the technique to a researcher to ensure proper execution. Second, participants were provided with a daily acupressure log sheet to record their sessions and sign off each day, and these logs were reviewed weekly by the researchers. Third, daily SMS reminders were sent to encourage consistent performance of the acupressure sessions. Finally, during weekly follow-up visits, researchers checked the condition of the acupressure patches. In cases where a patch had detached, the participant was contacted and the researcher personally visited to reattach it, ensuring the continuity of the intervention.

### 2.5 Instruments

#### 2.5.1 Sleep subjective indicators

For the assessment of subjective sleep quality and quantity, the Pittsburgh Sleep Quality Index (PSQI), originally developed by Buysse et al. (1989) (19) and translated into Korean by Sohn et al. (2012) (20), was used. The PSQI is a widely used self-report questionnaire assessing sleep quality over a one-month interval, comprising 19 self-reported items. These items categorize into seven components: ‘subjective sleep quality’ (1 item), ‘sleep latency’ (2 items), ‘sleep duration’ (1 item), ‘habitual sleep efficiency’ (3 items), ‘sleep disturbances’ (9 items), ‘use of sleeping medication’ (1 item), and ‘daytime dysfunction’ (2 items). Each item is scored on a 4-point scale (0–3 points) and summed by component. The total global PSQI score, ranging from 0 to 21, is obtained by summing these seven component scores. Higher scores indicate poorer sleep quality, with a score greater than 5 suggesting significant sleep difficulties (20). The Cronbach’s α for the original instrument was 0.83 (19), and for the Korean version, it was 0.84 (20). The Cronbach’s α for this study was 0.72. 2.5.2 Sleep Objective indicators

#### 2.5.2 Sleep objective indicators

Polysomnography (PSG), the gold standard for assessing sleep status, was utilized to objectively measure sleep quality indices in this study (21). PSG recordings were conducted using Alice PDx (Respironics Inc., USA) before the start and after the end of the eight-week auricular acupressure therapy. PSG captured data through electroencephalography (EEG), electrooculography (EOG), and eye movements. The sleep-related variables derived from PSG included nine items: Total Sleep Time (TST), Sleep Onset Latency (SOL), Wake After Sleep Onset (WASO), Sleep Efficiency (SE), Non-Rapid Eye Movement Sleep Stage 1 (N1), Non-Rapid Eye Movement Sleep Stage 2 (N2), Non-Rapid Eye Movement Sleep Stage 3 (N3), Rapid Eye Movement (REM), and Slow-Wave-Sleep (SWS) (21). Definitions for these variables are as follows: TST is the total time spent in sleep; SOL is the time taken to fall asleep after lights out; WASO is the total minutes awake after initial sleep onset; SE is the percentage of time spent sleeping while in bed; SWS refers to the time spent in N3 out of total time in bed; and N1, N2, N3, and REM represent the ratio of each sleep stage to total sleep time (21).

For measurement, the researcher visited each subject in person between 6 p.m. and 8 p.m. to explain the PSG procedure. Eight electrodes were attached to the sides of both eyes, behind both ears, the center and back of the head, and above and below the subjects’ chin. Subjects were informed about examination precautions and instructed to remove the electrodes the following morning upon waking. The recorded data were analyzed through a specialized sleep analysis system, and interpreted values were used for examination.

#### 2.5.3 Blood glucose

In this study, the researcher measured postprandial blood glucose once a week using a portable glucose meter (Accu-Check Guide, Roche, Germany). Postprandial blood glucose was consistently measured two hours after the start of the meal, from before the experiment began until eight weeks after its conclusion. To enhance measurement accuracy, the finger for blood glucose testing was disinfected with a sterile alcohol cotton pad and thoroughly dried before measurement. Additionally, sufficient blood flow in the capillaries was encouraged prior to blood collection to prevent measurement errors. When measuring postprandial blood glucose, subjects were instructed to refrain from consuming anything other than their meals within two hours from the start of the meal. 2.5.4 Stress Subjective indicators Stress Subjective indicators

The Perceived Stress Scale (PSS), developed by Cohen et al. (1983) (22), was used to measure perceived stress in this study. The PSS consists of 10 items, each scored on a 5-point Likert scale (0=Never, 1=Almost never, 2=Sometimes, 3=Fairly often, 4=Very often). Total scores range from 0 to 40, with higher scores indicating higher perceived stress levels. Positive questions (items No. 4, 5, 7, and 8) were reverse-coded. The Cronbach’s α for the original scale by Cohen et al. (1983) was 0.84 (22). For the Korean version, translated by Park and Seo (2010) (23), the Cronbach’s α was 0.85 (23). The Cronbach’s α for this study was 0.78. 2.5.5 Stress Objective indicators

#### 2.5.4 Stress objective indicators

In this study, electroencephalography (EEG) was used as an objective indicator to measure stress. EEG measurements were performed using Neuroharmony, a computer program developed by the Korea Institute of Psychiatry based on a physiological perspective to measure changes in brain waves (24). For accurate EEG measurement, subjects were instructed to remove metal accessories before the test and wear the device on their head while sitting comfortably. Their EEG was measured for approximately 15 minutes, during which subjects were encouraged to breathe comfortably to maintain a stable state. Measurements were performed in a comfortable environment to minimize external influences.

Analysis of the measured EEG data was performed to derive specific brain function indices and stress-related metrics. The stress index was calculated by dividing the ratio of delta (0.5–3 Hz), theta (4–7 Hz), and high beta (30 Hz or higher) waves, separately for the left and right brain. Higher stress index scores indicate higher physical and mental stress levels. Conversely, the anti-stress index (referred to as stress resistance in the results section) was derived from these brainwave patterns, where a higher score indicates a better ability to cope with stress.

### 2.6 Date collection procedure

The researcher completed an academic course in auricular acupressure expertise authorized by the Korea-China Naturopathy Association and obtained a relevant certificate before conducting the study. Participant recruitment was completed from April to June 2024. Subjects were recruited by requesting cooperation from the managers of four elderly welfare facilities in Seoul, Incheon, and Changwon, Korea, and by posting recruitment notices at each facility. Following trial registration (KCT0009524, registered June 13, 2024), the 8-week auricular acupressure intervention was implemented from June 15 to August 10, 2024, including pre- and post-intervention assessments. Written consent was obtained from all subjects who voluntarily agreed to participate. Patients with type 2 diabetes were recruited based on the predefined selection and exclusion criteria.

The researcher directly applied auricular acupressure to the ears of the subjects in both the intervention and control groups. A single-blind study method was implemented to prevent subjects from knowing their group assignment. Postprandial blood glucose levels were measured weekly for eight weeks using a mobile blood glucose meter whenever subjects visited a senior welfare facility for auricular acupressure application.

Harms were defined as any physical discomfort, adverse skin reactions (e.g., irritation, redness, or allergic responses), dizziness, or pain associated with auricular acupressure application. Harms were systematically assessed at each weekly visit through direct inquiry from the researcher and by spontaneous participant reporting.

At baseline (preliminary survey), electroencephalography, polysomnography, questionnaires, and postprandial blood glucose measurements were carried out. The general and health-related characteristics of the subjects were investigated. When necessary, the researcher directly read and recorded the questions while subjects filled out the questionnaires. After eight weeks of the treatment, a post-survey was conducted. Electroencephalography, polysomnography, questionnaires, and postprandial blood glucose measurements were performed in the same way as the pre-survey. Additionally, subjects were instructed to continue their existing diabetes medication to prevent the intervention of exogenous variables and to notify the researcher of any medication changes. They were also instructed to maintain their usual eating and activity patterns.

### 2.7 Ethical considerations

The study was conducted after obtaining the approval from the Institutional Review Board of Ewha Womans University (IRB NO. ewha- 202403-0025-01) to protect the subjects ethically. It was explained to the subjects that all information and data collected from them would be kept confidential and would not be used for any purpose other than research. In addition, the subjects were informed that they could withdraw their consent to the study at any time without any disadvantages and that all related data would be deleted.

### 2.8 Data analysis

The collected data were analyzed using the IBM SPSS Statistics 26.0. The specific analysis method is as follows

1. The general and health-related characteristics of the intervention and control groups were analyzed through descriptive statistics using mean, standard deviation, frequency, and percentage.
2. The prior homogeneity test on the general and health-related characteristics between the intervention group and control group was analyzed through independent t-test, Fisher’s exact test, and chi-squared test.
3. The normality test for the dependent variables of the intervention group and control group before the intervention was analyzed through the Shapiro-Wilk test, and the nonparametric statistic was used for the variables that did not follow the normal distribution.
4. The intra- and inter-group differences and time-dependent changes in the outcome variables of the intervention and placebo control group were analyzed using Generalized Estimating Equations (GEE), the Mann-Whitney U test, paired t-test, and independent sample t-test.
5. All statistical significance levels were set at *p* <.05.

## 3 Result

No adverse events were reported during the intervention period. All participants completed the eight-week auricular acupressure protocol without any complaints, injuries, or discontinuation related to the intervention.

### 3.1 Participant characteristics

The general and health-related characteristics of the subjects were investigated through a preliminary survey. Gender, age, education, and economic level were examined for general characteristics, whereas exercise status, smoking and drinking, snacking, duration of diabetes, diabetes medications taken, insulin use, hospitalization for diabetes, hypoglycemia, diabetic complications, status of other diseases, and use of complementary and alternative therapies were assessed for health-related characteristics.

Independent t-test, Fisher’s exact test, and chi-squared test were performed to verify the prior homogeneity between the intervention and control groups. As shown in **Table 1**, it was confirmed that the two groups were homogeneous.

**Table 1:** General characteristics and homogeneity test between intervention and control groups.

(N = 42)					
Variable	Characteristics	Intervention (n=21)	Control (n=21)	κ^2^ or t	p
		M ± SD or n (%)			
Age	(yr)	76.90 ± 5.13	75.95 ± 7.69	0.472	0.640
Height	(cm)	157.10 ± 9.04	155.95 ± 8.96	0.412. 0.200	0.683 0.843
Weight	(kg)	60.67 ± 8.83	61.33 ± 12.47		
DM	(yr)	10 ± 2.501	16.381 ± 2.805	0.008 0.107 2.593	0.931 0.743 0.107
Sex	Male	8(38.10%)	6 (28.6%)		
	Female	13(61.90%)	15 (71.4%)		
Graduate	≥elementary	9(42.9%)	7 (33.3%)		
	≥middle	1 (4.8%)	7 (33.3%)	6.467	0.091
	≥high	3 (14.3%)	7 (33.3%)		
	≥college	8 (38.1%)	–		
Economic	Upper	1 (4.8%)	4 (19.0%)	6.467	0.091
	Middle	10 (47.6%)	14 (66.7%)	0.200	0.843
	Lower	9 (42.9%)	3 (14.3%)		0.454^†^ 0.454^†^
Exercise	No	3 (14.3%)	6 (28.6%)		
	Yes	18 (85.7%)	15 (71.4%)		0.488^†^ 0.488^†^
Smoking	No	21 (100%)	19 (90.5%)		
	Yes	–	2 (9.5%)		1.000^†^ 1.000^†^
Drinking	No	18 (85.7%)	17 (81.0%)		
	Yes	3 (14.3%)	4 (19.0%)		0.751^†^ 0.751^†^
Snack	No	9 (42.9%)	7 (33.3%)		
	Yes	12 (57.1%)	14 (66.7%)	0.008	0.931
Medication	No	–	–	0.000	1.000
	Yes	21 (100%)	21 (100%)		
Insuline	No	21 (100%)	20 (95.2%)		1.000^†^
	Yes	–	1 (4.8%)		
Admission	No	21 (100%)	19 (90.5%)		0.488^†^
	Yes	–	2 (9.5%)		
Hypo	No	21 (100%)	18 (85.7%)		0.232^†^
	Yes	–	3 (14.3%)		
Complications	No	21 (100%)	18 (85.7%)		0.232^†^
	Yes	–	3 (14.3%)		
Disease	No	5 (23.8%)	6 (28.6%)		1.000^†^
	Yes	16 (76.2%)	15 (71.4%)		
Other use	No	20 (95.2%)	21 (100%)		1.000^†^
	Yes	1 (4.8%)	–		

^†^Fisher’s exact test

According to the results shown by the homogeneity test between the intervention and control groups for the dependent variables before the intervention (**Table 2**), there was no statistically significant difference between the two groups in all variables. In particular, the values of the intervention and control group in the blood glucose test showed difference resulting in 156.52 ± 34.52 and 170.95 ± 57.37, respectively; however, such difference was due to the high scores in certain subjects of the control group. Therefore, it was confirmed that the distribution of the data was homogeneous in the nonparametric test of the control and intervention group (Z=-0.189, *p* = 0.850), implying that the statistical homogeneity was tested.

**Table 2:** Testing for normality and homogeneity of the dependent variable between the intervention and control groups prior to intervention.

(N = 42)								
Variable	Intervention (n=21)			Control (n=21)			t or Z	p
	M ± SD	Shapiro-Wilk		M ± SD	Shapiro-Wilk			
		s	p		s	p		
Stress	15.38 ± 4.79	0.9387	0.2058	17.43 ± 3.26	0.9416	0.2345	-1.619	0.113
PSQI	8.71 ± 3.54	0.8654	0.0079	9.05 ± 2.38	0.9324	0.1541	-0.889^†^	0.374^†^
BST	156.52 ± 34.52	0.9736	0.8113	170.95 ± 57.37	0.8473	0.0038	-0.189^†^	0.850^†^
Brain wave								
Lt.Physical	52.49 ± 20.95	0.961	0.544	32.17 ± 15.82	0.919	0.082	1.023	0.318
Rt.Physical	52.82 ± 21.70	0.738	0.738	36.03 ± 16.08	0.935	0.171	1.758	0.192
Lt.mental	1.84 ± 0.68	0.939	0.210	1.18 ± 0.55	0.854	0.005	1.463^†^	0.234^†^
Rt.mental	1.73 ± 0.62	0.976	0.850	1.21 ± 0.49	0.902	0.038	2.792^†^	0.162^†^
Lt,against	34.57 ± 21.18	0.966	0.635	57.05 ± 18.84	0.893	0.026	-3.157^†^	0.657^†^
Rt,against	34.73 ± 22.16	0.963	0.585	52.68 ± 19.26	0.914	0.066	0.578	0.451
Pulse wave								
LF	5.24 ± 1.11	0.8412	0.0030	5.08 ± 1.51	0.9440	0.2609	0.529^†^	0.605^†^
HF	5.02 ± 0.97	0.9412	0.2300	4.98 ± 1.07	0.8869	0.0197	0.327^†^	0.752^†^
LF/HF	1.08 ± 0.18	0.9367	0.1876	1.01 ± 0.23	0.9460	0.2856	1.043	0.303
Polysomnography								
TST	300.85 ± 86.92	0.8601	0.0064	284.35 ± 135.88	0.9283	0.1271	-0.113^†^	0.919^†^
SE(%)	57.63 ± 18.70	0.9138	0.0653	58.60 ± 24.57	0.9051	0.0439	-0.616^†^	0.546^†^
SOL	223.90 ± 119.25	0.8597	0.0063	217.93 ± 141.99	0.9352	0.1752	0.415^†^	0.687^†^
TIB	524.75 ± 55.79	0.9727	0.7922	502.28 ± 131.54	0.7675	0.0002	0.150^†^	0.889^†^
WASO	161.48 ± 93.43	0.8745	0.0116	191.29 ± 148.17	0.8947	0.0277	-0.213^†^	0.840^†^
N1	89.95 ± 44.67	0.9576	0.4683	74.74 ± 43.57	0.9618	0.5540	1.117	0.270
N2	171.80 ± 60.41	0.9107	0.0568	174.63 ± 113.03	0.9358	0.1800	-0.101	0.920
N3	15.95 ± 20.66	0.7833	0.0004	12.31 ± 15.71	0.7840	0.0004	0.239^†^	0.815^†^
REM	21.12 ± 20.47	0.8655	0.0079	20.76 ± 29.31	0.7345	0.0001	0.440^†^	0.661^†^
SWS	9.31 ± 12.92	0.738	<0.001	11.38 ± 14.66	0.792	<0.001	-0.404^†^	0.687^†^

^†^Mann-Whitney U test, SD: Standard deviation

Lt. Physical: Left Physical Stress/Rt. Physical: Right Physical Stress/Lt. Mental: Left Mental Stress/Rt. Mental: Right Mental

Stress/Lt. Against: Left Against Stress/Rt. Against: Right Against Stress/TST: Total Sleep Time/SE (%): Sleep Efficiency/

SOL: Sleep Onset Latency/TIB: Time in Bed/WASO: Wake After Sleep Onset/N1: Non-Rapid Eye Movement Sleep Stage 1/

N2: Non-Rapid Eye Movement Sleep Stage 2/N3: Non-Rapid Eye Movement Sleep Stage 3/REM: Rapid Eye Movement Sleep/

SWS, Slow-Wave Sleep

The Shapiro-Wilk test was used to test the normality of the dependent variables of the study (**Table 2**). The results of the test showed that some variables did not follow a normal distribution. The variables that followed the normal distribution were PSS, left/right brain physical stress index, right brain stress resistance, N1, and N2, and parametric tests were applied to them. For other variables that did not follow the normal distribution in either intervention or control group, a nonparametric test was applied.

### 3.2 Sleep

This study evaluated the effect of auricular acupressure on sleep quality in the elderly patients with type 2 diabetes through PSQI and PSG. As a result of the analysis, a partial significant improvement was observed in sleep. The SWS, which indicates the level of deep sleep, increased significantly by 12.64% in the intervention group (|t|=2.585, *p* = 0.001), and the difference from the control group was also significant (|t|=3.046, *p* < 0.001, *d* = -0.90). Significant differences were also observed in SE, which indicates sleep efficiency (|t|=2.354, *p* = 0.019, *d* = 0.68). The WASO and SOL, which imply sleep continuity and onset, also showed significant differences between the two groups (|t|=2.692, *p* = 0.007, *d* = -0.56; |t|=2.027, *p* = 0.043, *d* = 0.70). There was also a significant difference in the ratio of N2 sleep stage, which indicates changes in sleep structure (|t|=2.027, *p* = 0.043,d=0.50).

No significant differences were observed between the two groups in total sleep time (TST), N1, N3, ratio of REM sleep, and subjective sleep measures (PSQI). These results are shown in **Table 3**.

**Table 3:** Differences in changes of dependent variables between intervention and control groups.

(N = 42)								
Variable	Group	Pretest mean ± SD	Posttest mean ± SD	Change mean ± SD	Within t or Z	P-value within	Between t or Z	P-value between Cohen’s d (*d*)
stress	Intervention	15.38 ± 4.79	14.38 ± 5.84	-1.00 ± 6.22	0.737	0.470	-0.382	0.705
	Control	17.43 ± 3.26	15.62 ± 7.59	-1.81 ± 7.46	1.111	0.280		(*0.12*)
PSQI	Intervention	8.71 ± 3.54	7.71 ± 2.53	-1.00 ± 2.81	1.630	0.119	-1.371	0.170
	Control	9.05 ± 2.38	7.33 ± 2.80	-1.71 ± 2.80	2.810	0.011		(*0.25*)
Lt_Physical	Intervention	52.49 ± 20.95	25.99 ± 13.21	-26.50 ± 26.35	4.608	<0.001	4.246	<0.001
	Control	32.17 ± 15.82	35.44 ± 17.32	3.26 ± 18.37	-.814	0.425		(*-1.31*)
Rt_Physical	Intervention	52.82 ± 21.70	25.92 ± 12.90	-26.90 ± 26.33	-5.539	<0.001	3.983	<0.001
	Control	36.03 ± 16.08	37.63 ± 16.56	1.60 ± 19.54	0.126	0.901		(*-1.23*)
Lt_mental	Intervention	1.84 ± 0.68	0.97 ± 0.58	-0.87 ± 0.75	-3.547	<0.001	-3.870	<0.001
	Control	1.18 ± 0.55	1.37 ± 0.59	0.19 ± 0.69	-1.420	0.156		(*-1.47*)
Rt_mental	Intervention	1.73 ± 0.62	0.99 ± 0.47	-0.73 ± 0.76	-3.360	<0.001	-3.837	<0.001
	Control	1.21 ± 0.49	1.42 ± 0.52	0.21 ± 0.65	-1.789	0.074		(*-1.33*)
Lt_against	Intervention	34.57 ± 21.18	66.66 ± 15.06	32.09 ± 26.28	-3.696	<0.001	-4.122	<0.001
	Control	57.05 ± 18.84	55.00 ± 18.94	-2.05 ± 22.49	-0.795	0.427		(*1.40*)
Rt_against	Intervention	34.73 ± 22.16	66.74 ± 14.58	32.01 ± 25.81	-3.771	<0.001	-3.769	<0.001
	Control	52.68 ± 19.26	53.11 ± 18.99	0.43 ± 24.93	-0.966	0.334		(*1.24*)
LF	Intervention	5.24 ± 1.11	5.03 ± 1.05	-0.21 ± 1.60	-0.383	0.702	-0.088	0.930
	Control	5.08 ± 1.51	4.85 ± 1.65	-0.23 ± 0.86	-0.932	0.351		(*0.02*)
HF	Intervention	5.02 ± 0.97	4.58 ± 0.80	-0.44 ± 1.23	-1.682	0.093	-1.337	0.181
	Control	4.98 ± 1.07	4.91 ± 1.09	-0.06 ± 0.74	-0.155	0.877		(*-0.37*)
LF_HF	Intervention	1.08 ± 0.18	1.11 ± 0.22	0.04 ± 0.26	-0.667	.512	-0.968	0.339
	Control	1.01 ± 0.23	0.98 ± 0.26	-0.03 ± 0.18	0.742	0.467		(*0.31*)
TST	Intervention	300.85 ± 86.92	342.06 ± 112.46	41.21 ± 162.24	-1.442	0.149	-1.850	0.064
	Control	284.35 ± 135.88	274.89 ± 149.27	-9.46 ± 148.52	-0.672	0.501		(*0.33*)
SE	Intervention	57.63 ± 18.70	64.37 ± 18.30	6.74 ± 28.25	-1.234	0.217	-2.354	0.019
	Control	58.60 ± 24.57	47.00 ± 24.73	-11.60 ± 25.55	-2.068	0.039		(*0.68*)
_SOL	Intervention	223.90 ± 119.25	181.72 ± 86.72	-42.18 ± 150.44	-0.973	0.330	-2.027	0.043
	Control	217.93 ± 141.99	288.43 ± 148.24	70.50 ± 171.55	-1.655	0.098		*(-0.70*)
TIB	Intervention	524.75 ± 55.79	523.79 ± 72.29	-0.97 ± 96.66	-0.365	0.715	-0.906	0.365
	Control	502.28 ± 131.54	563.32 ± 81.06	61.04 ± 158.83	-1.913	0.056		(*-0.47*)
WASO	Intervention	161.48 ± 93.43	129.07 ± 85.54	-32.40 ± 126.75	-1.338	0.181	-2.692	0.007
	Control	191.29 ± 148.17	241.58 ± 138.75	50.29 ± 167.18	-1.293	0.196		(*-0.56*)
N1	Intervention	89.95 ± 44.67	79.14 ± 48.48	-10.81 ± 53.75	0.922	0.368	1.124	0.268
	Control	74.74 ± 43.57	80.98 ± 54.16	6.24 ± 44.04	-0.649	0.524		(*-0.35*)
N2	Intervention	171.80 ± 60.41	224.72 ± 95.32	52.92 ± 134.10	-1.222	0.236	-2.027	0.043
	Control	174.63 ± 113.03	168.43 ± 119.13	-6.20 ± 100.14	0.284	0.779		(*0.50*)
N3	Intervention	15.95 ± 20.66	19.88 ± 23.71	3.93 ± 32.93	-0.436	0.663	-0.508	0.612
	Control	12.31 ± 15.71	12.48 ± 17.17	0.17 ± 22.36	-0.105	0.917		(*0.13*)
REM	Intervention	21.12 ± 20.47	18.17 ± 20.98	-2.95 ± 26.36	-0.608	0.543	-0.202	0.840
	Control	20.76 ± 29.31	15.00 ± 20.80	-5.76 ± 34.54	-0.628	0.530		(*0.09*)
SWS	Intervention	9.31 ± 12.92	21.95 ± 21.534	-12.64 ± 22.66	-2.585	0.001	-3.046	<0.001
	Control	11.38 ± 14.66	8.33 ± 11.542	3.047 ± 9.893	-1.352	0.176		(*-0.90*)

Lt. Physical: Left Physical Stress/Rt. Physical: Right Physical Stress/Lt. Mental: Left Mental Stress/Rt. Mental: Right Mental

Stress/Lt. Against: Left Against Stress/Rt. Against: Right Against Stress/TST: Total Sleep Time/SE (%): Sleep Efficiency/

SOL: Sleep Onset Latency/TIB: Time in Bed/WASO: Wake After Sleep Onset/N1: Non-Rapid Eye Movement Sleep Stage 1/

N2: Non-Rapid Eye Movement Sleep Stage 2/N3: Non-Rapid Eye Movement Sleep Stage 3/REM: Rapid Eye Movement Sleep/

SWS, Slow-Wave Sleep

### 3.3 Glucose

Repeated measures analysis revealed a significant group × time interaction for blood glucose changes (F = 4.73, *p* = 0.032), indicating differential patterns between groups over the study period. A significant time effect was also observed (F = -6.50, *p* = 0.019), while the group effect approached significance (F = 6.83, *p* = 0.668).

As shown in **Table 4**, both groups had similar baseline blood glucose levels (intervention: 173.81 ± 45.39 mg/dL vs. control: 170.95 ± 57.37 mg/dL). However, divergent patterns emerged throughout the intervention period. The intervention group demonstrated a consistent downward trend, with blood glucose levels decreasing from baseline (173.81 mg/dL) to post-intervention (125.95 ± 35.40 mg/dL), representing a substantial within-group reduction of 47.86 mg/dL. Conversely, the control group showed a progressive increase from baseline (170.95 mg/dL) to post-intervention (206.62 ± 105.09 mg/dL), indicating a deterioration of 35.67 mg/dL.

**Table 4:** Changes in blood glucose levels in experimental and control groups.

(N = 42)					
Bst	Intervention (n=21)	Control (n=22)	source^†^	F^†^	P^†^
	M ± SD or n (%)				
Pre	173.81 ± 45.39	170.95 ± 57.37			
1 weeks	165.24 ± 43.61	181.29 ± 68.29	Group	6.83	0.668
2 weeks	167.71 ± 53.83	189.62 ± 91.37			
3 weeks	160.24 ± 45.30	179.67 ± 60.21			
4 weeks	165.67 ± 52.03	177.19 ± 52.98	Time	-6.50	0.019
5 weeks	150.00 ± 43.67	183.67 ± 73.55			
6 weeks	145.90 ± 36.76	191.00 ± 83.77			
7 weeks	135.90 ± 31.40	196.71 ± 91.25	Time *Group	4.73	0.032
Post	125.95 ± 35.40	206.62 ± 105.09			

**Figure 3:**
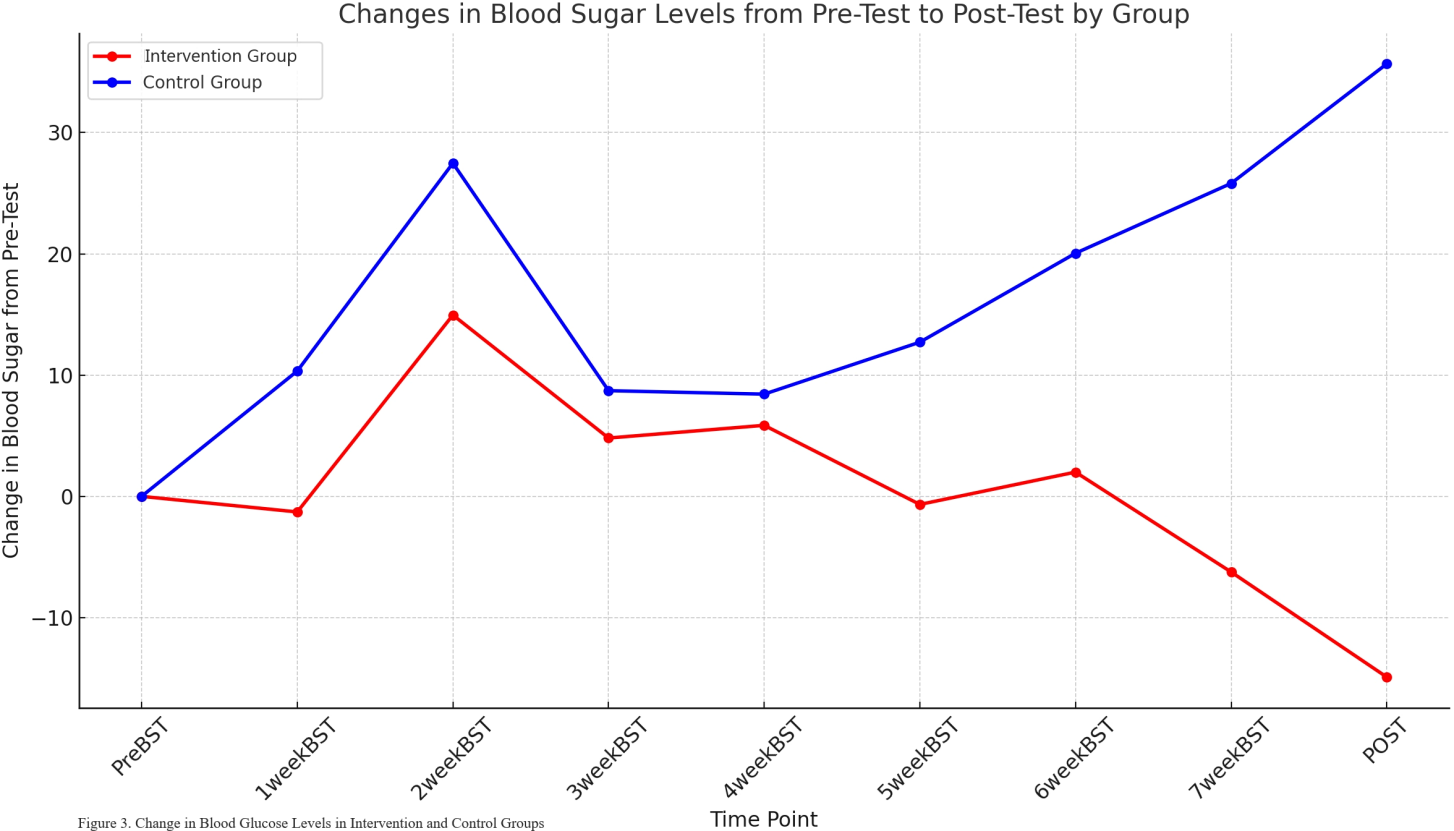
Changes in postprandial blood glucose levels from pre-test to post-test by group. This line graph displays the mean change in postprandial blood glucose levels (mg/dL) from baseline (PreBST) over eight weeks of intervention for both the intervention (red line) and Control (blue line) groups. The x-axis represents the time points of measurement (PreBST, 1weekBST,…, 7weekBST, POST), and the y-axis indicates the change in blood glucose from the pre-test value.

The between-group difference became increasingly pronounced over time, with the most notable divergence observed from week 5 onwards (intervention: 150.00 ± 43.67 mg/dL vs. control: 183.67 ± 73.55 mg/dL). By study completion, the intervention group achieved a final mean blood glucose of 125.95 ± 35.40 mg/dL compared to 206.62 ± 105.09 mg/dL in the control group. The overall trend of blood glucose changes throughout the study period is illustrated in **Figure 3**.

### 3.4 Stress

For the stress-related variables, electroencephalography (physical/mental stress, stress resistance) and the Perceived Stress Scale (PSS) were used to comprehensively evaluate the subjective experience of stress response and brain activity response. The results of the analysis are presented in **Table 3**.

Significant changes were observed in objective stress index. Physical stress in the intervention group significantly decreased in both left brain (|t|=4.608, *p* < 0.001) and right brain (|t|=5.539, *p* < 0.001). Mental stress also significantly decreased in the left brain (|t|=3.547, *p* < 0.001) and right brain (|t|=3.360, *p* < 0.001). The stress resistance significantly increased in both left brain (|t|=3.696, *p* < 0.001) and right brain (|t|=3.771, *p* < 0.001). On the other hand, there was no significant difference in subjective stress levels between the two groups.

## 4 Discussion

This study examined the effects of auricular acupressure on blood glucose, stress, and sleep in elderly patients with type 2 diabetes and sleep disorders. The results indicated that auricular acupressure was effective in reducing blood glucose and stress levels while improving sleep quality in this population. This study sought to address limitations in prior research by utilizing advanced objective measures, including polysomnography (PSG) and electroencephalography (EEG), to provide a deeper understanding of the underlying physiological mechanisms.

The PSG analysis revealed substantial improvements in key sleep parameters, most notably a 12.64% increase in slow-wave sleep (SWS) duration in the intervention group (p=0.001), with significant between-group differences (p<0.001). Additionally, sleep efficiency improved significantly (p=0.019), while wake after sleep onset and sleep latency showed meaningful reductions (p=0.007 and p=0.043, respectively). The N2 sleep stage ratio also demonstrated significant improvement (p=0.043). These findings have important clinical implications for elderly patients with type 2 diabetes, as SWS is crucial for bodily recovery, metabolic regulation, and enhancing insulin sensitivity (6). The improvements in both N2 and SWS stages are particularly meaningful for this population, as these sleep stages are often compromised with aging and their reduction is linked to various health risks (5, 25). The enhanced sleep efficiency and reduced sleep fragmentation observed suggest that auricular acupressure may help restore more physiologically beneficial sleep patterns in elderly diabetic patients.

These findings align with previous research demonstrating beneficial effects of auricular acupressure on sleep in older adults. Lee et al. (2025) (12) reported improvements in objective sleep measures in older adult patients with T2D, while Suen et al. (2002) (10) found improvements in nighttime sleep duration and sleep efficiency. However, our study advances this field by employing PSG, which provides detailed insights into complex sleep architecture that cannot be captured by activity trackers used in previous studies (21, 26, 27). This deeper physiological assessment suggests that auricular acupressure modulates sleep structure at a fundamental neurophysiological level, potentially through vagal nerve pathways that influence brainstem sleep-wake regulation centers (14, 28). Our objective PSG results provide strong neurophysiological evidence supporting the clinical applicability of auricular acupressure for sleep management in elderly T2D patients.

An important finding was the discrepancy between objective and subjective sleep measures. While PSG demonstrated clear physiological improvements, both groups showed similar improvements in subjective sleep quality (PSQI scores: intervention group 8.71 to 7.71; control group 9.05 to 7.33), with no between-group differences. This pattern suggests that elderly patients may not immediately perceive physiological sleep improvements, highlighting the critical importance of objective measures in evaluating interventions for this population. This discordance has been reported in previous auricular acupressure studies, with Yoon (2024) (29) and Jun et al. (2024) (27) noting that objective measures consistently demonstrate stronger effects than subjective assessments. The observed subjective improvements in both groups likely reflect non-specific study effects, as elderly participants often show improvements when receiving attention and care through research participation (26). This finding underscores the value of PSG in capturing subtle but clinically meaningful physiological changes that may precede subjective awareness. Future research should explore this gap, potentially with longer follow-up periods, to assess the sustainability of objective improvements and their eventual subjective perception.

The EEG analysis revealed substantial reductions in both physical and mental stress markers across bilateral brain regions. Physical stress decreased significantly in both hemispheres (left: p<0.001; right: p<0.001), with large between-group effect sizes (left: d = -1.31; right: d = -1.23). Mental stress showed similar patterns (left: p<0.001, d = -1.47; right: p<0.001, d = -1.33), while stress resistance improved markedly (bilateral p<0.001, d = -1.24). These neurophysiological changes, reflected in the appropriate regulation of delta, theta, and high beta waves, indicate improved stress coping abilities and reduced fatigue and tension (30, 31). The large effect sizes suggest substantial therapeutic benefits that may contribute to better diabetes management, as chronic stress is known to impair glucose regulation and insulin sensitivity. Interestingly, these objective neurophysiological improvements were not reflected in subjective stress measures (PSS), consistent with findings by Bae and Kim (2021) (30) and Kim and Jung (2011) (31). This pattern suggests that neurophysiological stress reduction may precede subjective awareness, similar to the sleep findings.

Additionally, heart rate variability (HRV) parameters were assessed as complementary objective stress indicators. Both groups showed decreases in HF and LF components, with no significant between-group differences (HF: p=0.181; LF: p=0.930). These findings contrast with previous studies that reported significant HRV improvements following auricular acupressure interventions in younger populations (30). The lack of between-group differences in our elderly participants may reflect adaptation to repeated testing environments or non-specific relaxation effects from study participation, as previously observed in elderly populations undergoing physiological assessments (26). This suggests that multi-modal stress assessment using various physiological measures may be necessary to comprehensively evaluate intervention effects, as neurophysiological measures (EEG) demonstrated more pronounced changes than cardiovascular autonomic indicators in our study.

Regarding blood glucose control, repeated measures analysis revealed a significant group × time interaction (F = 4.73, p=0.032), indicating differential patterns between groups over the study period. The intervention group demonstrated a consistent decline from baseline (173.81 mg/dL) to post-intervention (125.95 mg/dL), representing a clinically meaningful reduction of 47.86 mg/dL, while the control group showed a progressive increase from 170.95 mg/dL to 206.62 mg/dL. The difference in blood glucose changes between the intervention and control groups emerged from week 4. At week 4, mean blood glucose decreased to 162.38 mg/dL in the intervention group and increased to 179.38 mg/dL in the control group. By week 8, blood glucose in the intervention group decreased to 141.62 mg/dL, showing a significant decrease compared to the initial level.

These findings, particularly the significant reduction in blood glucose in the intervention group, are consistent with previous studies. Fasting blood glucose was significantly reduced in older patients after 6 weeks of auricular acupressure treatment in a prior study (8), and after a 12-week intervention in patients aged 50 years and older (9). This consistency suggests that auricular acupressure is an effective complementary intervention in managing type 2 diabetes. Regarding the mechanism, Feng et al. (2018) (28) suggested that auricular acupressure can regulate the balance of the autonomic nervous system and affect insulin sensitivity. The temporary increase in blood glucose observed at weeks 2 and 4 in this study could be attributed to initial changes in insulin sensitivity, with the subsequent decrease reflecting a gradual restoration of autonomic nervous system balance.

The observed improvements across multiple physiological domains suggest that auricular acupressure may work through central nervous system pathways, potentially via vagal nerve connections to brainstem structures involved in sleep-wake regulation, stress response, and metabolic control (14, 28). However, the precise neurophysiological mechanisms underlying these observed benefits remain to be elucidated and require direct investigation through future studies incorporating neuroendocrine indicators such as cortisol, catecholamines, and comprehensive autonomic function assessments. Future mechanistic research should incorporate comprehensive neuroendocrine markers including salivary or serum cortisol levels, catecholamine measurements, and detailed autonomic nervous system assessments to directly examine hypothalamic-pituitary-adrenal axis involvement and validate the proposed central nervous system regulatory pathways.

Several limitations should be considered. While this study demonstrates the potential effectiveness of acupressure interventions, our compliance measures were primarily qualitative. Future studies would benefit from incorporating detailed quantitative compliance metrics such as session completion rates and patch adherence duration. Additionally, as participants were recruited from specific welfare institutions within South Korea, expanding recruitment to more diverse geographical and demographic populations would enhance the generalizability of these findings. Future studies should comprehensively assess the effect of auricular acupressure on insulin metabolism by measuring insulin resistance indices (e.g., HOMA-IR) and insulin secretion function (e.g., C-peptide). It is important to set a longer study period to examine long-term glycemic control and stabilization, considering potential changes in insulin resistance. This multifaceted assessment will provide a clearer picture of when the effects of auricular acupressure can be stabilized and its mechanisms of long-term glycemic control.

These findings provide valuable insights into the neurophysiological interactions between stress, sleep, and metabolic control in diabetic patients and highlight the clinical applicability of non-pharmacological interventions for comprehensive diabetes management in elderly populations.

## 5 Conclusions

This randomized, sham-controlled, single-blind clinical trial demonstrated that eight weeks of auricular acupressure therapy effectively improved multiple physiological parameters in elderly patients with type 2 diabetes, including objective sleep quality, neurophysiological stress markers, and blood glucose control. Since auricular acupressure is non-invasive and has few side effects, it holds strong potential for clinical application in the elderly. It can serve as an effective complementary and alternative therapy for stress, sleep, and blood glucose management and may be utilized in various community settings, such as elderly care facilities and health centers, contributing to a comprehensive approach to chronic disease management in this vulnerable population.

## Data Availability

The raw data supporting the conclusions of this article will be made available by the authors, without undue reservation.
